# "Scientific trends of pharmacological methods in labor pain management: bibliometric analysis of epidural, spinal, and other approaches, 2000–2025"

**DOI:** 10.1590/1806-9282.20251401

**Published:** 2026-05-01

**Authors:** Remziye Gültepe

**Affiliations:** 1 Çanakkale Onsekiz Mart University, Faculty of Medicine, Department of Midwifery – Çanakkale, Turkey.

**Keywords:** Labor pain, Epidural analgesia, Spinal anesthesia, Analgesia, obstetrical, Bibliometric analysis, Delivery, obstetric

## Abstract

**OBJECTIVE:**

Labor pain management is a fundamental aspect of obstetric care. Pharmacological methods such as epidural and spinal analgesia are widely preferred in this process, but scientific productivity and trends regarding these approaches have not been sufficiently analyzed. The aim of this study was to provide a bibliometric assessment of the scientific profile in the field of pharmacological obstetric pain management by examining the literature published between 2000 and 2025.

**METHODS:**

Web of Science Core Collection database was searched with the keywords "labour pain management," "childbirth pain," "labour analgesia," "obstetric analgesia," "epidural analgesia," "spinal anaesthesia," "pharmacological analgesia," and "pharmacological pain management." The distribution of publications by years, contributions by countries and institutions, collaboration networks, most cited studies, and journal distribution were analyzed.

**RESULTS:**

A total of 1,606 publications were analyzed. Scientific productivity in the field of epidural and spinal analgesia has increased significantly, especially after 2019. The vast majority of publications are based in the USA, the UK, and China. The most cited publications focus on the efficacy, safety, and complications of pharmacological methods. Collaboration maps indicate a multidisciplinary and international research network.

**CONCLUSION:**

Pharmacological labor pain management literature reflects the current trends that guide clinical obstetric practice. When developing guidelines for the use of these methods in midwifery and obstetrics, qualitative aspects as well as the quantitative increase in the literature should be taken into account. Our study sheds light on the research gaps in this field and the research areas that can be focused on for the future.

## INTRODUCTION

Labor pain is a complex phenomenon requiring multidisciplinary approaches in the field of women's health and obstetric services^1^. In addition to the physiological dimension of pain, the psychological and sociocultural effects that shape the woman's birth experience cannot be ignored^2^. The severity of pain perceived by women in labor interacts with many factors including fear, anxiety, lack of information, and level of social support^3^.

In modern obstetrics, many pharmacological and non-pharmacological methods have been developed to manage labor pain. Epidural and spinal analgesia stand out as the most commonly used methods of pharmacological pain control in labor^4^. While epidural analgesia positively affects women's labor preferences and satisfaction because it provides effective pain control, there are ongoing discussions that it may prolong the second stage of labor and increase the rates of intervention delivery. Although spinal anesthesia is a method preferred especially in cesarean deliveries, it is also used in normal deliveries in certain clinical situations^5^.

Despite the clinical effectiveness of epidural and spinal analgesia, the debate surrounding their potential risks and the uneven global access to such interventions highlights an important problem in maternal care. In many low- and middle-income countries, pharmacological pain relief options remain limited due to infrastructural, economic, and workforce barriers, leading to inequalities in women's birth experiences^3^. Moreover, the dominance of clinical trials evaluating safety and maternal–neonatal outcomes has overshadowed broader questions about the evolution of research in this field, such as the geographic distribution of studies, the most influential authors, and the patterns of international collaboration^4,5^. This lack of systematic mapping of the literature creates a gap in understanding how evidence has accumulated, where knowledge clusters exist, and which areas remain underexplored.

In recent years, a significant increase has been observed in the literature on the use of pharmacological methods. A large number of studies have been conducted, especially on epidural and spinal analgesia, from different aspects including clinical safety data, patient satisfaction, and maternal and neonatal outcomes^6,7^. However, bibliometric data such as in which areas these publications are concentrated, which countries are pioneers, and how research collaborations are shaped have not yet been systematically analyzed.

Bibliometric analyses are valuable tools in health sciences for identifying literature trends and research gaps^8^. In midwifery and obstetric care, they help develop evidence-based roadmaps to guide clinical practice.

This study aims to reveal publication trends, citation dynamics, cross-country collaborations, and research gaps by examining scientific publications on pharmacological labor pain management between 2000 and 2025. Our study is an important resource for updating field-specific guidelines, developing midwifery education curricula, and increasing multidisciplinary collaborations.

## METHODS

This bibliometric study examined publications on pharmacological labor pain management (including epidural and spinal anesthesia, opioid analgesics such as morphine and fentanyl, nitrous oxide, and other pharmacological methods such as inhalational agents and local anesthetic infiltration) published between 2000 and 2025.

Data were collected from the Web of Science Core Collection between June 1 and 15, 2025. The literature search was limited to the Web of Science database, which comprehensively covers high-quality journals and bibliometric data, which is standard practice in bibliometric analysis.

The following keywords were used in the search: "*Labor pain management," "Childbirth pain," "Obstetric analgesia," "Labor analgesia," "Epidural analgesia," "Spinal analgesia,"* and "*Pharmacological pain management."* Searches were conducted in titles, abstracts, and keyword fields.

### Inclusion/exclusion criteria

Studies published between 2000 and 2025 in English, focusing on pharmacological methods for labor pain management (including epidural, spinal, opioid analgesia, nitrous oxide, and other pharmacological interventions) and categorized as original research, narrative reviews, systematic reviews, or meta-analyses were included. Studies on non-pharmacological methods, cesarean anesthesia, non-full-text documents (e.g., abstracts, letters), and duplicates were excluded. The literature search was limited to the Web of Science database due to its comprehensive coverage of high-quality journals and bibliometric data.

### Data collection and analysis

**Figure 1:**
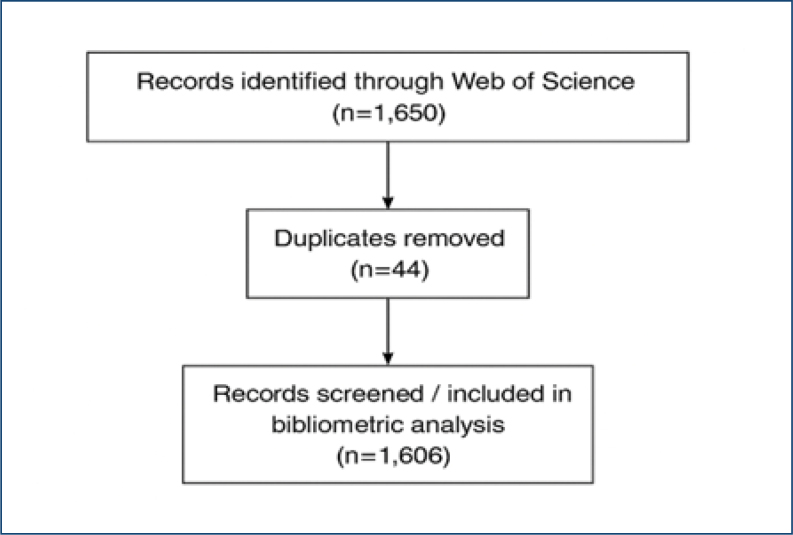
PRISMA 2020 flow diagram illustrating the literature search and selection process. A total of 1,650 records published between 2000 and 2025 were identified through Web of Science. After removal of duplicate records (n=44), 1,606 publications were included in the bibliometric analysis.

Key bibliometric data (author, year, country, institution, journal, citations, H-index, collaborations, keywords, and methods) were extracted. Analyses were conducted using VOSviewer (v1.6.20) for collaboration and keyword mapping, the Bibliometrix R package (v4.2.1) for citation, publication type, and trend analyses, and Microsoft Excel for data cleaning and frequency calculations. Temporal trends in publication output were examined descriptively and visualized in Figure 1; given the bibliometric nature of the study, formal statistical tests for significance were not applied.

## RESULTS

A total of 1,650 records published between 2000 and 2025 were identified through Web of Science. After removing duplicate records (n=44), 1,606 publications were included in the bibliometric analysis. The literature search and selection process is presented in Figure 1.

Of the 1,606 publications, the majority were original research articles (n=1,443; 89.9%), followed by narrative reviews (n=102; 6.4%) and systematic reviews/meta-analyses (n=61; 3.8%). Regarding pharmacological methods, epidural analgesia was the most frequently studied approach (58.3%), followed by spinal anesthesia (21.6%) and combined spinal–epidural (CSE) or other pharmacological methods (20.1%) (Table 1).

**Table 1:** Distribution of article types and pharmacological methods (2000–2025).

Characteristic		n	%
Article type			
	Original research	1,443	89.9
	Narrative review	102	6.4
	Systematic review/meta-analysis	61	3.8
Pharmacological methods			
	Epidural analgesia	936	58.3
	Spinal anesthesia	347	21.6
	Combined spinal–epidural and other methods	323	20.1
Total		1,606	100

**Figure 2:**
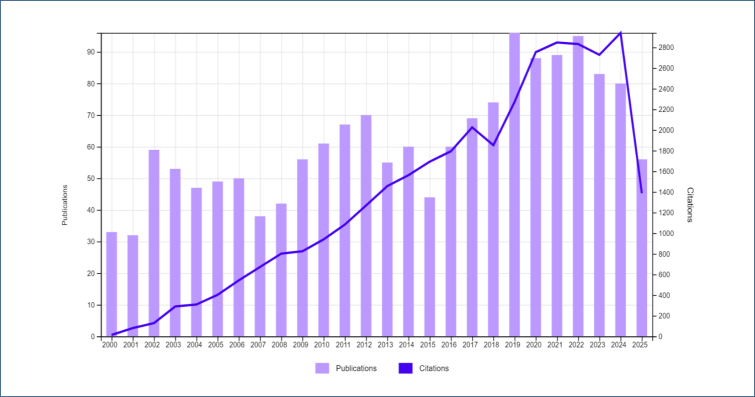
Number of articles and citations published on pharmacological management of labor pain between 2000 and 2025..

An increase in the number of publications was observed, especially since 2019, with the highest number of publications reached in this year (n=96) (Figure 2). Given the bibliometric nature of the study, formal statistical significance testing was not applied, but the trend is clearly illustrated in the figure.

The trend indicates a growing research focus on pharmacological methods in childbirth. The majority of publications were original research (89.91%), reflecting a strong emphasis on empirical data production in the field.

Among the most prolific authors, C.A. Wong (n=15), R.J. McCarthy (n=13), T. Ghi (n=12), L.C. Tsen (n=12), and C.F. Weiniger (n=12) stand out. The majority of the publications were published in high-impact journals such as American Journal of Obstetrics & Gynecology (2024 IF ≈ 8.4), Obstetrics & Gynecology (2024 IF=4.7), and Birth: Issues in Perinatal Care (2024 IF=2.5).

It is seen that well-established institutions such as Harvard University, University College London, and Peking University make important contributions within the scope of international collaborations. At the country level, the USA leads with 28.27% of publications, followed by the UK, China, Israel, and Australia. This indicates that research is largely concentrated in countries with advanced health systems (Table 2).

**Table 2:** Most productive countries and scientific impact indicators for publications on pharmacological management of labor pain (2000–2025).

Rank	Country	Number of publications	Number of citations	H-index
1	United States of America (USA)	454	12.493	58
2	England	145	3.675	35
3	China	117	1.055	17
4	Israel	105	1.469	22
5	Australia	96	2.018	27

The publications received 20,667 citations, with an average of 22.11 citations per article. Table 3 presents the top five most cited studies, led by Hodnett et al.'s (2002) systematic review on women's satisfaction with labor pain management (677 citations). Other influential works include studies by Zhang et al. (2010), Waldenström et al. (2004), Pan et al. (2004), and Leighton et al. (2002), highlighting the key references shaping the field.

**Table 3:** Most influential studies in the literature: most cited publications (2000–2025).

Rank	Author(s)	Year of publication	Journal name	Number of journal citations	Title	Key findings
1	Hodnett ED	2002	American Journal of Obstetrics and Gynecology	677	Pain and women's satisfaction with the experience of childbirth: a systematic review	It has been shown that women's experience of pain in childbirth is closely related not only to physiological but also to psychological satisfaction.
2	Zhang J et al.	2010	Obstetrics and Gynecology	541	Contemporary patterns of spontaneous labor with normal neonatal outcomes	The study defined current labor patterns and revealed that the use of an epidurals may prolong the course of labor but does not affect neonatal outcomes.
3	Waldenström U et al.	2004	Birth-Issues in Perinatal Care	473	A negative birth experience: prevalence and risk factors in a national sample	The study examined the prevalence and risk factors for adverse birth experiences and concluded that pain management during birth significantly affects women's experiences.
4	Pan PH et al.	2004	International Journal of Obstetric Anesthesia	243	Incidence and characteristics of failures in obstetric neuraxial analgesia and anesthesia: a retrospective analysis of 19,259 deliveries	The study analyzed failure rates in obstetric epidural/spinal analgesia and reported that technical factors played an important role in failure.
5	Leighton BL et al.	2002	American Journal of Obstetrics and Gynecology	234	The effects of epidural analgesia on labor, maternal, and neonatal outcomes: a systematic review	It has been demonstrated that epidural analgesia is the most effective method in reducing labor pain, but its effect on maternal and neonatal outcomes is limited.

Table 3 presents the top five most cited studies, with key findings indicating that women's satisfaction with childbirth is closely tied to pain management strategies, epidural analgesia remains the most effective pharmacological method, and negative birth experiences are common but influenced by multiple risk factors.

The most frequently encountered terms in the keyword analysis were "epidural analgesia," "labour pain," and "maternal outcomes." This shows that publications in the field of pharmacological management of labor pain are focused on both clinical effects and patient satisfaction. The findings clearly demonstrate the importance and continuity of the subject in the academic literature.

## DISCUSSION

This study examined the scientific literature published between 2000 and 2025 on the pharmacological management of labor pain using a bibliometric method. The findings reveal a growing academic interest in labor analgesia, particularly since 2019. This increase is associated with the standardization of obstetric analgesia in clinical practice, the prioritization of patient comfort and satisfaction in women's health policies, the development of medical technologies, and the strengthening of the role of anesthesia practitioners^9,10^.

Pharmacological labor pain management is mainly addressed under three headings: epidural analgesia, spinal analgesia, and CSE analgesia. In addition to these, other pharmacological strategies such as inhalation anesthetics, intravenous opioids, and patient-controlled analgesia (PCA) have been studied. Our bibliometric results show that epidural analgesia was the most frequently studied method (58.3%), confirming its recognition as the "gold standard" in obstetric clinics, especially in high-income countries^11,12^. This predominance can be explained by its effective analgesic effect, high maternal satisfaction, and adaptability with low-dose regimens that minimize complications^13,14^.

Spinal analgesia, although predominantly used in cesarean deliveries, was represented in 21.6% of the publications, reflecting its role in selected vaginal deliveries. CSE analgesia accounted for about 20.1% of the literature, underlining its growing but still technically limited application. These proportions in our bibliometric analysis align with clinical observations that epidural procedures dominate, while spinal and CSE remain supplementary^15^.

Although spinal analgesia is generally preferred in cesarean deliveries, it is also considered an option in short vaginal deliveries. However, it is emphasized that spinal analgesia requires meticulous patient selection due to the risks of complications such as hypotension and headache^15^. CSE analgesia combines the advantages of spinal with rapid onset and epidural with long-term pain control. However, the technical complexity of CSE applications limits its wide application in clinical practice^16^.

### Evaluation of pharmacological methods with sub-dimensions

This bibliometric analysis shows that pharmacological methods in the management of labor pain have been extensively studied in terms of efficacy, safety, side effect profile, patient satisfaction, birth outcomes, and economic dimensions.

- Effectiveness and safety: There is strong evidence in the literature that pharmacological analgesia significantly reduces labor pain^17^. However, complications such as maternal hypotension, urinary retention, motor block, and fever are still important research topics^18^.
- Neonatal effects: There are different results in the literature regarding the effects of epidural and spinal analgesia on neonatal Apgar scores, umbilical artery gases, and breastfeeding onset. Recent studies show that especially low-dose opioid combinations may reduce neonatal effects^19^.
- Effects on the labor process: Although some studies have reported that epidurals prolong the second stage, this has been shown to be minimized with modern low-dose techniques. The increase in operative delivery rates such as forceps or vacuum remains a controversial issue^20^.
- Maternal satisfaction and psychosocial dimension: The psychological effects of pharmacological methods on the birth experience are also frequently examined in the literature. It has been reported that epidurals reduce the fear of labor and may prevent posttraumatic stress symptoms in women^21^. However, some studies also reveal that not being able to completely control the labor process creates dissatisfaction in some women. Moreover, recent evidence highlights the importance of evaluating long-term psychosocial outcomes such as postpartum pain sensitivity, breastfeeding success, and mother–infant bonding, which remain underexplored in current literature^22^.
- Economic and access dimension: While epidural and spinal analgesia are widely used in developed countries, access is still limited in low- and middle-income countries. This situation creates a global inequality in access to healthcare services. Lack of anesthesiologists, equipment costs, and cultural factors increase this limitation^23^.

### Global trends and research gaps

Bibliometric findings show that publications on pharmacological management of labor pain are largely concentrated in high-income countries. The USA, UK, China, Israel, and Australia are among the countries with the highest scientific production in this field. In contrast, the number of publications on the subject is limited in low- and middle-income regions such as Africa, Southeast Asia, and South America. This situation is not only related to a lack of scientific interest but also to structural inequalities in health systems. For example, multinational maternal health data from the World Health Organization reveal that the use of pharmacological analgesia at birth in these regions is less than 4%^24^. Furthermore, in societies with high socioeconomic inequalities, racial and ethnic minorities have been shown to benefit less from neuraxial analgesia^25^.

In addition to structural inequalities, cultural attitudes also play a critical role. In some societies, pharmacological methods are perceived as disrupting the naturalness of childbirth, leading to a preference for non-pharmacological strategies^23^. Such cultural factors highlight the importance of context-specific guidelines and further qualitative research to assess acceptability and applicability.

However, the existing literature provides limited data not only on the efficacy and safety profiles of pharmacological methods, but also on long-term psychobiological outcomes such as postpartum pain sensitivity, lactation success, and mother–infant attachment. In this context, there is a need for further research that holistically assesses not only acute pain management but also effects extending into the postpartum period.

### Strengths and limitations of the study

The strength of this study is that it provides a comprehensive bibliometric review over a wide time period (2000–2025). However, only the Web of Science database was used, and only English-language publications were included. This may lead to the exclusion of some important studies. In addition, bibliometric analyses do not assess the qualitative content of research; therefore, it is recommended to supplement with systematic reviews.

Another limitation concerns the quality of the evidence. The publications included in the bibliometric dataset vary in methodological quality, and the heterogeneity of study designs limits the comparability of outcomes. In addition, citation-based metrics are inherently vulnerable to bias, as studies with positive results are more likely to be published and cited than those with negative or inconclusive findings. These factors should be taken into account when interpreting bibliometric trends.

## CONCLUSION

This bibliometric analysis demonstrates that pharmacological methods—particularly epidural analgesia—dominate the scientific literature on labor pain management between 2000 and 2025. A marked increase in publications since 2019, the leading role of high-income countries such as the USA, UK, and China, and the influence of a small group of prolific authors underline the concentrated yet unequal nature of the research field. While the clinical effectiveness and maternal satisfaction associated with neuraxial techniques are well-established, persistent gaps remain regarding access disparities, cultural applicability, neonatal and long-term maternal outcomes, and integration of patient preferences. Addressing these gaps through multidisciplinary, cross-country, and culturally sensitive research will be crucial to shaping more equitable and patient-centered obstetric analgesia practices worldwide.

## Data Availability

The datasets generated and/or analyzed during the current study are available from the corresponding author upon reasonable request.
